# Copper homeostasis and cuproptosis in myocardial infarction: molecular mechanisms, treatment strategies and potential therapeutic targets

**DOI:** 10.3389/fphar.2025.1525585

**Published:** 2025-09-26

**Authors:** Zhen Shen, Zhichao Liu, Siqi Cai, Huanjie Fu, Yongkang Gan, Xiaofeng Li, Xizhen Wang, Chang Liu, Wenjia Ma, Jinhong Chen, Ningcen Li

**Affiliations:** ^1^ Department of Clinical Laboratory, Affiliated Hospital of Shandong Second Medical University, Weifang, Shandong, China; ^2^ School of Rehabilitation Medicine, Shandong Second Medical University, Weifang, Shandong, China; ^3^ College of Art, Nanjing University of Information Science and Technology, Nanjing, Jiangsu, China; ^4^ Department of Cardiovascular, Second Teaching Hospital of Tianjin University of Traditional Chinese Medicine, Tianjin, China; ^5^ Department of Vascular Surgery, Tianjin Academy of Traditional Chinese Medicine Affiliated Hospital, Tianjin, China; ^6^ Research Center of Experimental Acupuncture Science, Tianjin University of Traditional Chinese Medicine, Tianjin, China

**Keywords:** copper, cuproptosis, myocardial infarction, mitochondrion, molecular mechanisms

## Abstract

Copper (Cu), an essential trace element for normal bodily functions, plays a pivotal role in cardiac muscle biology and is critical for cardiac function and metabolism. Recent research increasingly links Cu-related cell death (cuproptosis) to diseases like myocardial infarction (MI). Cu overload drives cuproptosis via mitochondrial dysfunction, lipoylated protein aggregation, and Fe-S cluster reduction, inducing proteotoxic stress and linking inflammatory/ROS pathways to MI progression. Therefore, it can be hypothesized that cuproptosis is a novel therapeutic target for MI. In this review, we explore the primary molecular mechanisms, treatment strategies and potential therapeutic targets involved in cuproptosis. Moreover, the insights obtained from this review provide a novel perspective on the pathogenesis of MI and new targets for its intervention.

## 1 Introduction

Myocardial infarction (MI), characterized by the high morbidity and mortality rates, remains a significant challenge in global healthcare, and continues to claim lives (Roth et al., 2020). The development of atherosclerotic plaques in the coronary vessel wall, which can lead to vessel stenosis or potential rupture, constitutes the primary mechanism behind MI through thrombotic vessel occlusion (Doenst et al., 2019). Importantly, myocardial cell death plays a crucial role in MI pathogenesis. The deceased cardiomyocytes can not be replaced by viable ones due to their limited regenerative capacity (Addis and Epstein, 2013). Intervention of myocardial cell death is vital for improving MI prognosis. Immediate coronary blood flow restoration, namely, the early success in reperfusion treatment, is the cornerstone for minimizing myocardial injury and enhancing MI patient outcomes (Xiang et al., 2024). Currently, electrocardiogram results and high-sensitivity troponin measurements are the preferred tests for MI evaluation and diagnosis. Nevertheless, they have inherent limitations in the detection of mild myocardial injury, early MI, and stable coronary artery disease (Bhatt et al., 2022). Therefore, identifying effective cardiac biomarkers and therapeutic targets for MI will significantly contribute to its early diagnosis and improved prognosis.

The cell death mode in MI has become an important point of research. While apoptosis and necrosis are the primary forms of cell death, cuproptosis, the novel programmed cell death form resulting from copper (Cu), was identified in 2022 (Kahlson and Dixon, 2022). Cu plays a vital role as a micronutrient in various physiological processes across nearly all cell types. However, the excessive intracellular Cu level may cause oxidative stress while disrupting cell functions, emphasizing the significance of maintaining Cu homeostasis (Zhong et al., 2022). Cuproptosis mainly occurs when intracellular Cu binds to lipoylated components in the tricarboxylic acid (TCA) cycle. The process results in Cu-bound lipoylated mitochondrial protein aggregation, therefore reducing iron–sulfur (Fe–S) clusters, inducing proteotoxic stress, and finally resulting in cell death (Tsvetkov et al., 2022). Recently, cuproptosis has attracted considerable attention for its involvement in the pathophysiology of tumors, Menkes disease, Wilson disease, neurodegenerative diseases, and other conditions (Chen et al., 2022). Furthermore, research on its role in cardiovascular disease is advancing steadily. Therefore, this review focuses on recent research progress concerning cuproptosis in MI and discusses its vital role in MI modulation.

## 2 The physiological functions of copper

Cu, an essential trace element essential for maintaining normal bodily functions, exists in organisms in two forms: Cu ions (Cu^1+^, reduced form) or Cu ions (Cu^2+^, oxidized form). Both forms are essential for numerous physiological responses in the human body (Chen et al., 2020). The dietary Cu dose recommended for adults is 0.9 mg/day, and the overall body Cu content is estimated to be approximately 100 mg (Bost et al., 2016).

The Cu level in cells is modulated through specific absorption systems, which can be balanced by ion-activated P-type ATPases associated with Cu elimination. The absorption and efflux transporters are also important for maintaining Cu homeostasis (Abeyrathna et al., 2020). The Cu content remains relatively stable in the body; the insufficient free Cu level can impair metal-binding enzyme function, while excess free Cu level can cause cellular damage and even death (Wang et al., 2023). To shield cells against free Cu-induced detrimental impacts, the sophisticated intracellular metallochaperone system has evolved. These proteins acquire Cu from donor proteins, facilitate its uptake, and transport it to specific cellular sites through direct interactions with target proteins. This mechanism ensures the acquisition of necessary Cu cofactors and mitigates adverse effects in association with elevated free Cu levels (Fukai et al., 2018).

In addition to its general physiological functions, Cu plays a pivotal role in cardiac muscle biology, significantly impacting cardiac function and metabolism (Mohammadifard et al., 2019). It catalyzes reactions in various physiological events influencing mitochondrial energy generation (Tian et al., 2023), neurotransmitter and tyrosine metabolism (Campolo et al., 2014), redox homeostasis (Méndez et al., 2024), and extracellular matrix remodeling (Liu et al., 2024). Studies have shown that Cu is not only a risk factor for MI but also serves as a specific biomarker for numerous biological processes. It acts as a cofactor for antioxidant enzymes, participates in glycosylation, influences cytochrome and mitochondrial activity, and modulates vascular responses to inflammatory stimuli. Disruption of Cu homeostasis, either through deficient or excess Cu level, can perturb these critical processes, potentially exacerbating disease progression (Lim et al., 2023). Cu is closely associated with MI, both as a causative factor and as a consequence.

## 3 Molecular mechanisms

### 3.1 Angiogenesis enhancement

Myocardial regeneration and angiogenesis are important for the MI pathogenic mechanism, serving as the fundamental mechanisms for cardiac function restoration post-infarction. In particular, angiogenesis occupies a central role in creating a rejuvenated microenvironment under ischemic conditions, which can therefore promote myocardial rejuvenation (Wu et al., 2021). Hypoxia-inducible factor 1 (HIF-1) is a primary transcription factor governing angiogenesis. It oversees oxygen delivery by controlling angiogenesis and vascular remodeling, and regulates oxygen utilization through glucose metabolism and redox homeostasis (Semenza, 2014). In prolonged MI, the activity of HIF-1 is chiefly modulated by HIF-1α, which serves as a crucial subunit of HIF-1. Cu has a regulatory role in numerous aspects of HIF-1, such as the stabilization of HIF-1α, the assembly of transcription complexes, and the binding to the hypoxic response element sequences present in the target genes (Chen et al., 2011). In addition, it also contributes to the selective regulation of HIF-1 binding to the target angiogenic genes, thus influencing Cu-dependent angiogenic factor expression (Martin et al., 2005; Xiao et al., 2020). Studies have shown that during myocardial ischemic injury, Cu depletion results in the deactivation of HIF-1-regulated angiogenesis, causing the upregulation of genes like vascular endothelial growth factor (VEGF) related to angiogenesis (Zhang et al., 2018; Sato and Takeda, 2023). Cu supplementation can enhance the HIF-1 transcriptional activity, restore angiogenic capacity, and increase capillary density in the heart. Dietary Cu supplementation has been found to replenish cardiac Cu, stimulate HIF-1 activity, upregulate VEGF expression, and accelerate angiogenesis, while reversing hypertrophic cardiomyopathy in mice (Jiang et al., 2007). Li et al. found a gradual decrease in heart Cu level over time in a MI mouse model, while serum Cu levels increased (Li et al., 2018). Furthermore, prolonged ischemia is correlated with the decreased Cu content in ischemic heart, significantly inhibiting angiogenesis (Kobayashi et al., 2017). Considering the crucial role of Cu in promoting angiogenesis, increasing Cu concentration in the heart can effectively reactivate angiogenesis in ischemic myocardium, which can provide a potential alternative therapeutic approach for MI.

### 3.2 Oxidative stress activation

In physiological situations, cells are under the oxidation-antioxidant defense balance. Oxidative stress is triggered upon the disruption of the balance, causing cell damage and the onset of different disorders. During both ischemia-reperfusion in acute MI and the subsequent chronic remodeling phase, oxidative stress significantly induces cardiac injury. Reactive oxygen species (ROS) produced in mitochondria exert a vital role in mechanisms contributing to ischemia-reperfusion injury, including induction of mitochondrial permeability transition and oxidative injury to intramitochondrial molecules and structures. Apart from acute settings, mechanisms such as extracellular remodeling, inflammatory signal transduction, and pro-apoptotic signaling facilitating post-infarction remodeling, can be modulated through mitochondrial ROS (Bugger and Pfeil, 2020). Normally, the lower ROS levels can be balanced by detoxification mechanisms, which are critical for cellular signaling, excitation-contraction coupling, gene expression regulation, cell proliferation, migration, apoptosis, and differentiation. This process, which is known as redox signaling, involves the special yet reversible oxidation/reduction modifications of signal transduction components in cells. Under pathological conditions, ROS may result in oxidative modifications of key cell macromolecules such as lipids, DNA and proteins, affecting subcellular organelles including the mitochondria, sarcolemma, nucleus, and sarcoplasmic reticulum (Dubois-Deruy et al., 2020). It has been reported in some studies that, excessive Cu may trigger an oxidative stress response (Yang et al., 2019). As a transition metal element, Cu participates in Fenton-like reactions, which are essential in a variety of diseases (Pi et al., 2023). Cu ions which are redox-active catalyze Fenton reactions, generating ROS (Zhou et al., 2022). These ions have convertible valences, serving as the active centers in Fenton-like reactions. Cu^2+^ catalyzes the generation of oxygen from the overexpressed H_2_O_2_ and combating hypoxia, while Cu^1+^ catalyzes H_2_O_2_ to produce hydroxyl radicals and other highly toxic ROS. Cu^2+^ reacts with glutathione (GSH) to form GSSH, which can produce against the generated ROS and induce cell apoptosis (Fu et al., 2021). Zhou et al. (2022) demonstrated that Cu exposure induces ROS-mediated NF-κB activation in microglia, which initially serves as a pro-survival signal by upregulating anti-oxidant genes. However, sustained Cu accumulation disrupts mitochondrial homeostasis via PINK1/Parkin inactivation, leading to mitophagy failure and NLRP3 inflammasome-dependent pyroptosis. Chen et al. (2021a) demonstrated that depletion of Cysteine-rich protein 2 (CRIP2) and Cu-induced degradation of CRIP2 increased ROS levels and induced autophagy in cancer cells. Mechanistically, Cu^1+^ promotes CRIP2 ubiquitination and proteasomal degradation, thereby relieving its suppressive effect on autophagy. This process is associated with increased ROS production, which triggers autophagic flux independent of the AMPKα-ULK1 pathway. Based on Zhu et al. (2023a), elevated Cu^2+^ induces neuronal and histopathological alterations in the mouse hypothalamus by generating excessive ROS, which triggers mitophagy and disrupts mitochondrial dynamics. The process is characterized by inhibited mitochondrial fusion (via downregulation of Mfn1/Mfn2) and enhanced fission (via upregulation of Drp1/FIS1), leading to mitochondrial swelling and cristae breakage. Furthermore, Cu is a vital element in Cytochrome c oxidase (CcO) and superoxide dismutase (SOD) 1, which are of great importance for mitochondrial energy metabolism and antioxidation capacity. Dietary Cu restriction leads to cardiac hypertrophy, eventually contributing to MI (Wen et al., 2022). These studies suggest that Cu ions damage cells and mitochondria by promoting ROS generation while promoting activation of oxidative stress-related pathways, exerting potential influence on the pathogenesis of MI.

### 3.3 Effect on myocardial fibrosis

In MI, ischemic cell death initiates the multiphase reparative response, in which the fibrotic scar predominantly formed by myofibroblasts and fibroblasts substitutes the injured tissue. This process can cause biochemical, geometrical, and biomechanical alterations in unaffected ventricular walls, triggering the reactive remodeling characterized by perivascular and interstitial fibrosis (Talman and Ruskoaho, 2016). After MI, cardiac fibroblasts experience dynamic phenotype transition regulating the inflammatory, angiogenic and reparative responses. This transition is initiated by danger-associated molecular patterns released from necrotic cells, such as HMGB1 and ATP, which bind to TLR4/6 on fibroblasts, activating the NF-κB pathway and upregulating pro-inflammatory cytokines (Prabhu and Frangogiannis, 2016). Necrotic cells can produce danger signals in an inflammatory stage during infarct healing, activating innate immune pathways and triggering the potent inflammatory response. Inflammatory signals promote leukocyte-endothelial cell adhesion, leading to the monocyte and neutrophil extravasation. Suppressing inflammatory response may be associated with the activation of reparative cells (Prabhu and Frangogiannis, 2016). Furthermore, after matrix debris and dead cells are cleared out of the infarct site, the transforming growth factor β (TGF-β) cascades and anti-inflammatory pathways are activated, causing fibroblast transformation into myofibroblasts expressing α-smooth muscle actin (α-SMA). After activation, myofibroblasts can produce abundant matrix proteins, contributing to forming the collagen-based scar for protecting infarcted ventricle against potential fatal events like cardiac rupture (Venugopal et al., 2022).

Actually, a study indicates that dietary Cu restriction induces mouse cardiac hypertrophy and failure, whereas Cu supplementation abolishes hypertrophy while preventing progression into heart failure. The restoration of normal cardiac function with Cu repletion may be caused by favorable downregulation of gene expression in myocardial tissue (Elsherif et al., 2004). By contrast, dietary Cu deficiency results in cardiac hypertrophy, fibrosis, and myofibril disarray. Wold et al. (2001) suggested that the impaired cardiac contractile function observed in Cu-deficient whole hearts might not stem from the depressed contractile function at the single-cell level but rather from cardiac fibrosis. Cu deficiency impairs lysyl oxidase activity, a Cu-dependent enzyme essential for collagen cross-linking, leading to disorganized collagen deposition and fibrotic heterogeneity. Another study shows that in the rodent models of cardiac hypertrophy, the Cu chelator Trientine can supplement Cu in the heart, thereby reducing cardiac fibrosis (Liu et al., 2018). Xiao et al. (Y et al., 2023) developed a MI model in rhesus monkeys through coronary artery ligation and employed the ultrasound-guided Cu albumin microbubble technology for targeted Cu delivery to ischemic myocardial tissue. This approach could significantly increase Cu concentrations in infarcted areas, facilitate the relaxation of the collagen cross-linking network, restore vascular density, and enhance cardiac contractility. These findings suggest that Cu inhibits fibroblast differentiation into myofibroblasts, promoting a profibrinolytic environment and therefore improving cardiac function. In conclusion, based on the aforementioned evidence, variations in Cu levels can influence the extent of cardiac fibrosis, potentially serving as a mechanism for ameliorating fibrotic heart disease.

### 3.4 Intervention in mitochondrial energy metabolism

Micronutrients like Cu make vital impacts on mitochondrial function, particularly in mitochondrial-rich tissues like cardiac muscles (Chen et al., 2011). COX11 and SCO1, key chaperones for CcO assembly, deliver Cu to the Cu(B) site of COX1 and Cu(A) site of COX2 subunits in the mitochondrial inner membrane, respectively, to facilitate CcO maturation (Horng et al., 2004). Mitochondria are the primary sites associated with energy generation, which are important for modulating different cell death types resulted from metal metabolism, immunotherapy, radiotherapy, and targeted antitumor therapies (Tian et al., 2023). Cu accumulates in the mitochondrial matrix to support the cuproenzyme maturation, like SOD and CcO. The transfer of Cu in the matrix can be facilitated through proteins belonging to the mitochondrial carrier family. Regulatory functions of Cu and resident cuproproteins in mitochondria are increasingly recognized to extend beyond the organelle itself. Mitochondrial Cu chaperones are implicated in modulating cellular Cu uptake and export, as well as promoting inter-organ communication (Cobine et al., 2021; Garza et al., 2023). Isei et al. (2021) found that hypoxic conditions diminished mitochondrial sensitivity to Cu, while increased Cu levels further stimulated the release of H_2_O_2_ from mitochondria during the oxidative metabolism of palmitoylcarnitine. In rats, a Cu deficient diet caused a 74% reduction in complex IV (Zuo et al., 2013). Zhang et al. (2020) demonstrated that Cu deficiency in diabetic hearts impairs myocardial function by down-regulating PGC-1α, a key regulator of mitochondrial biogenesis, and this defect is reversed by Cu chelation therapy. Specifically, Cu deficiency reduces the expression of mitochondrial Cu chaperones Cox17 and Cox11, leading to decreased CcO activity and mitochondrial ROS accumulation, which in turn suppresses PGC-1α expression and impairs mitochondrial biogenesis. Concurrently, changes of mitochondrial cristae and membranes disrupt the energy metabolism and contribute to myocardial damage (Chen et al., 2011; Halling and Pilegaard, 2020).

### 3.5 Regulation of lipid metabolism

Lipids and Cu are related to the pathogenic mechanisms of dyslipidemia-related disorders including obesity, neurological disorders, non-alcoholic fatty liver disease, and Wilson disease (the inherited disease characterized by Cu overload) (Muchenditsi et al., 2017; Lu et al., 2022; Qiu et al., 2023; Övermöhle et al., 2023). It is essential to comprehend the impact of Cu with lipid metabolism, aiming to identify potential therapeutic targets in conditions where Cu/lipid metabolism are disrupted. The same links may also exist in cardiovascular diseases with impaired Cu and lipid transport mechanisms. Genomic variances, especially in pathways involving coagulation and lipid metabolism, have been found in MI patients. Further knowledge of these risk factors, anatomical considerations, and pathophysiological processes can improve strategies for the prevention and treatment of MI in this patient population (Sagris et al., 2022).

**FIGURE 1:**
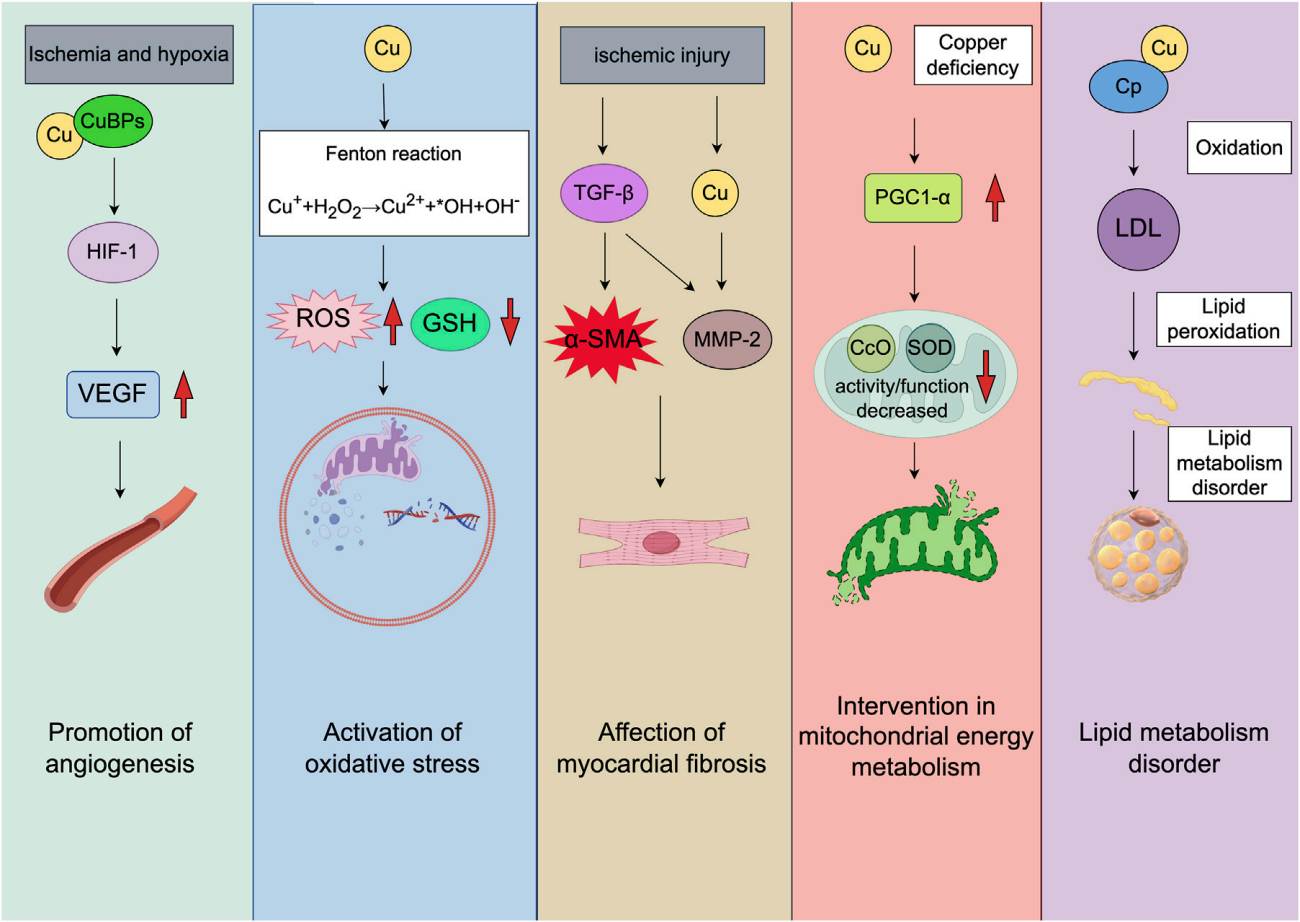
The molecular mechanisms of MI associated cuproptosis. The direction of the red arrow corresponds with increased or decreased molecules, respectively. The figure was created with Figdraw (https://www.figdraw.com/). CuBPs: copper-binding proteins; TGF-β: transforming growth factor-β; α-SMA: α-smooth muscle actin; MMP-2: matrix metalloproteinase 2; PGC1-α: peroxisome proliferator-activated receptor gamma coactivator 1-alpha; CcO: cytochrome c oxidase; SOD: superoxide dismutase.

Ceruloplasmin (Cp) is a predominant plasma protein containing 7 Cu atoms in each molecule, constituting 95% of circulating Cu among normal adult subjects (Fox et al., 2000). Its physiological roles include Cu transport, coagulation regulation, angiogenesis, defense against oxidative stress, and iron homeostasis (White et al., 2012; Linder, 2016). The increased serum Cp levels have been consistently observed in cardiovascular disorders including arteriosclerosis (Grammer et al., 2014), MI (Tang et al., 2012), and heart failure (Savic-Radojevic et al., 2017). Biochemical studies have revealed that Cp catalyzes low-density lipoprotein (LDL) oxidation *in vitro*, and the optimal activity is achieved in the presence of superoxide, which reduces the surface Cu atom of Cp (Fox et al., 2000). In addition, Wells et al. (2014) reported the positive correlations between Cu levels and triglycerides, whereas the negative correlations between Cu levels and high-density lipoprotein cholesterol levels. Blades et al. (2021) further clarified the tissue-specific roles of the Cu-regulating protein ATP7B, providing insights into the complex relationship between Cu and lipid metabolism. In summary, changes in Cu levels can indirectly influence lipid metabolism in the human body, potentially impacting the cardiovascular disease risk (Figure 1).

## 4 Clinical treatment strategies that target Cu-triggered cell death during MI

Micronutrients and trace elements are vital for the normal functioning of the body. Despite small amounts are required, alterations in their levels may result in serious diseases including MI and its consequences. In fact, one study (Begum et al., 2023a) demonstrates that the assessment of serum Cu levels in MI patients may be beneficial for taking appropriate measures to prevent free radical-induced reperfusion injury. Serum Cu shows potential significance for the prognosis and diagnosis of MI (Wen et al., 2022), with close associations being found in the alterations of serial serum Cu contents with markers including creatine phosphokinase and lactate dehydrogenase (Singh et al., 1985). Currently, researchers in the field of cardiovascular disease are investigating the correlation of Cu ion homeostasis with cardiovascular disease treatment. A summary of clinical studies on Cu and MI is provided in Table 1. Feng et al. (Feng et al., 2023) reported that Cu ions, combined with mild heat, promoted angiogenesis and significantly improved cardiac function while inhibiting ventricular remodeling. Natural antidotes can regulate Cu homeostasis and mitigate metal toxicity. Curcuminoids and their synthetic derivatives possess different medicinal properties, such as heavy metal chelation, antioxidation and anti-inflammation activities, cytotoxicity to cancer cells, and roles in hypertension management and apoptosis regulation. These properties highlight curcumin as a potential treatment for neurodegenerative diseases, cardiovascular diseases, malignancies, and conditions associated with metal overload (Maghool et al., 2023). Fang et al. (2024a) indicated that paeoniflorin (PF) significantly reduced the expression of Ferredoxin 1 and serum Cu levels, and increased the pyruvate levels. Moreover, PF can prevent from left ventricular remodeling following MI through mitigating myocardial cell apoptosis, inflammation, and fibrosis.

**TABLE 1:** Clinical studies on Cu homeostasis and myocardial infarction.

Author, year	MI cases	Research methodology	Result
Afridi et al. (2006)	193	Flame/graphite furnace atomic absorption spectrophotometer	The Zn/Cu ratios in the scalp hair (*p* < 0.01) of the MI group were significantly lower than those of the healthy groups
Guo et al. (2002)	34	Plasma spectrometer detection	Cu content increased
Kazi et al. (2008)	130	Flame atomic absorption spectrophotometry	In these subjects, the concentration of Cu was increased by 3.12% in the scalp hair and 22.5% in blood samples, respectively, when compared to those who survived a third MI attack
Khan et al. (1984)	27	Atomic absorption spectrometry	In AMI, the concentration of Cu was higher after 21–30 h (as compared with the values at 0–10 h)
Luo et al. (2002)	135	Plasma spectrometer detection	Cu content increased
Awadallah et al. (2006)	56	Turbidimetry and atomic absorption spectrophotometry	Serum concentrations of Cu were significantly higher in the MI group of patients than in the controls
Martín-Lagos et al. (1997)	12	Flame atomic absorption spectrophotometry for metal analysis in biological samples	The mean serum Cu level showed no statistical difference between MI patients (*p* > 0.05) and the healthy group. Male patients had no statistically different serum Cu values (*p* > 0.05) than female patients, and patient age had no statistical influence (*p* > 0.05) on serum Cu
Singh et al. (1985)	44	Sodium diethyldithiocarbamate method	A highly significant increase in serum Cu levels was observed in patients with AMI compared to those with angina and controls. Mean peak serum Cu levels were significantly higher (*p* < 0.001) in complicated cases of AMI than in uncomplicated cases
Taneja et al. (2000)	30	An atomic absorption spectrophotometer	Urine Cu levels in patients were approximately twofold lower; Cu in the urine of MI descendants was higher than that in patients (*p* < 0.001), but Cu in hair was lower in MI descendants compared to control counterparts (*p* < 0.001)
Versieck et al. (1975)	16	Neutron activation analysis	A statistically significant increase in serum Cu was observed after MI.
Khaki-Khatibi et al. (2018)	80	Atomic absorption spectrophotometry	Cu concentrations were higher in patients than in the control group (*p* < 0.001), indicating a positive diagnostic value for the disease
Tan et al. (1992)	138	Flame atomic absorption spectrophotometry (Varian Spectra AA-20)	The differences in serum Cu and Zn levels between patients and controls were magnified when the Cu/Zn ratios were calculated for both groups (*p* < 001)
Zama and Towns (1986)	71	X-ray fluorescence spectrometry and atomic absorption spectrometry	Significant differences (*p* < 0.001) in elemental levels were observed between the noninfarct and recent infarct groups, with the noninfarct group having higher cardiac levels of all three elements. Cardiac levels of Zn (p < 0.001) and Cn (*p* < 0.01) were significantly greater in the old-infarct group than in the recent-infarct group. Magnesium levels were higher in the recent-and-old-infarct group than in the recent infarct group (*p* < 0.01), suggesting elemental redistribution during MI to maintain myocardial integrity and function
Jain and Mohan (1991)	30	Colorimetric method of Ventura and King	Serum Cu levels increased from the first 24 h up to the 7th day after infarction, with a gradual decline that did not return to normal by the 14th day
Białkowska et al. (1987)	29	Neutron activation analysis for Zn and Cu concentrations	The Zn/Cu ratio in survivors of MI was significantly higher than in controls
Bakos et al. (1988)	30	Atomic absorption spectrometry	The mean values of serum Cu were lower in patients with AMI than in noncardiac patients
Shokrzadeh et al. (2009)	30	Atomic absorption spectrophotometry	The mean Cu level in the ISCMP group was significantly higher than that in healthy volunteers (*p* = 0.048)
Nowicki et al. (2021)	74	Inductively coupled plasma mass spectrometry (ICP-MS)	Higher concentrations of Cu, Zn, Mn, Co, and Fe were significantly associated with an increased risk of MI.
Begum et al. (2023b)	60	Serum Cu ion level detection	The mean serum Cu level was higher in the case group than in the control group: 105.44 ± 24.15 μg/dL vs 146.49 ± 23.52 μg/dL (*p* < 0.05)
Yesmin et al. (2016a)	60	Colorimetric method for serum Cu determination	The mean serum Cu level was significantly increased in AMI patients compared to the control group (*p* < 0.01)

### 4.1 Copper chelators

Chelation therapy is the option of treatment for Cu overload or intoxication. Various chelating agents are currently in clinical use, under investigation, or enter clinical trials. Obviously, chelation therapy has also been proposed as a treatment for certain neurodegenerative diseases and cardiovascular disorders (Tegoni et al., 2014). Tetrathiomolybdate (TM) is an orally active agent which can be used for disorders of Cu metabolism. TM works by chelating bioavailable Cu to form a tripartite TM-Cu-protein complex (Alvarez et al., 2010). Wei et al. (Wei et al., 2012) demonstrated that TM suppressed mouse atherosclerosis by decreasing vascular inflammation and bioavailable Cu without influencing iron homeostasis or oxidative stress. In addition, due to its Cu-dependent mechanism, TM may exert influence on angiogenesis (Brewer, 2005). Li et al. reported that TM exhibited significant anti-inflammatory properties by inhibiting Cu-dependent cytokines involved in inflammation. This anti-inflammatory effect may also contribute to the anticancer properties of TM, as cancer progression usually involves inflammatory cells and excessive angiogenic agents (Brewer, 2014). Other Cu complexes, including trientine, the Cu-aspirinate complex, and Cu (II) diethyldithiocarbamate, have shown potential in preventing and treating cardiovascular disorders (Zhu et al., 2023b). Based on clinical research, Cu^2+^-selective chelation with trientine is safe and well-tolerated, showing promise as a future therapeutic target for hypertrophic cardiomyopathy. The treatment shows efficacy in reducing left ventricular mass and myocardial fibrosis, while its high selectivity for Cu^2+^ avoids affecting serum zinc or iron levels, minimizing off-target toxicity. The ongoing TEMPEST trial will further validate its long-term safety and efficacy (Reid et al., 2022).

As the broad-spectrum metal-chelating agent, ethylenediaminetetraacetic acid disodium salt (EDTA) is explored due to its efficacy in treating MI (Ga et al., 2013). For example, according to one double-blinded, placebo-controlled trial (Escolar et al., 2020), disodium EDTA chelation therapy decreases the possibility of unfavorable cardiovascular outcomes among stable MI patients. However, concerns remain regarding its non-selective metal-binding profile, which may pose risks such as electrolyte imbalance or trace element depletion (Fulgenzi et al., 2020). While these findings warrant further exploration, the current evidence is insufficient to support routine clinical use of EDTA chelation for MI. Given the significant economic burden of cardiovascular diseases, definitive validation of this treatment’s safety and efficacy through large-scale randomized trials is essential before widespread recommendation.

### 4.2 Small-molecule inhibitors of copper chaperone proteins

ATOX1 is the significant Cu chaperone protein for mammalian cells. It can enhance genotoxic drug resistance by activating the DNA damage repair mechanisms (Jin et al., 2022). A growing body of evidence has also indicated that ATOX1 is vital for modulating cell migration, growth, autophagy, and apoptosis, and for organism development and reproduction (Yang et al., 2023a). Sudhahar et al. (2022) identified novel downstream nuclear ATOX1 targets related to ROS generation and inflammation, indicating that nuclear ATOX1 might be the candidate target used for treating inflammatory disorders like atherosclerosis.

Numerous studies are carried out on nanomedicine, which have led to the emergence of “nanocatalytic therapy,” in which catalytic responses regulated via nanomaterials can be used for the intervention with disease-related biomolecular processes (Kim et al., 2024). Exogenous nanomaterials often undergo rapid biotransformation once injected, which can impair their intended function. Notably, Cu-deposited ceria nanoparticles (CuCe NPs) have been reported to show promoted antioxidation efficacy when compared with pristine ceria nanoparticles. This is because that the released Cu buffers glutathione depletion and serves as the cofactor of SOD1. In MI models, CuCe NPs have exhibited therapeutic effects by improving perfusion and reducing tissue damage (Im et al., 2023). While CuCe NPs show promising therapeutic effects in preclinical ischemic models, clinical translation requires validation through large-scale trials. Further studies are needed to evaluate their long-term safety and define optimal delivery strategies for targeted accumulation in ischemic tissues.

Applying Cu ion chelators can reduce Cu ion contents, potentially causing severe toxicities that break essential physiological processes requiring Cu. The compound DCAC50 has been reported to specifically inhibit tumor cell growth exerting no influence on healthy cells by limiting intracellular Cu ion delivery while binding to Cu chaperone proteins CCS and ATOX1 (Karginova et al., 2019). Cu chelators show non-specific chelation with additional metal cations, causing subsequent adverse reactions. Based on studies on the mechanism underlying DCAC50, it suppresses Cu SOD1 activity, which depends on the cofactor Cu ions, through the interference with Cu ion transport, increase in ROS contents, influence on mitochondrial function, and the reduction of ATP generation (Inkol et al., 2020). Moreover, these findings can inform the development of anti-MI novel drugs.

### 4.3 Copper ionophore

Cu ionophores, also known as Cu transport-related drugs, enhance the bioavailability of Cu in cells. For instance, the Cu ionophore elesclomol selectively transports Cu^2+^ from the extracellular environment into mitochondria, where it is reduced to Cu^1+^, therefore generating ROS (Ge et al., 2022; Zheng et al., 2022). While Cu ionophores can address Cu deficiency by delivering Cu to small molecules within cells, they also pose a risk of increasing intracellular Cu levels, which may result in cell death.

Elesclomol, a well-known Cu ionophore with selectivity of tumor cells, is evaluated by clinical studies for cancer therapy (Zheng et al., 2022). Nevertheless, the precise mechanism underlying the selectivity of elesclomol is unclear, and thus further research is warranted to determine whether this selectivity can be leveraged to develop other Cu ionophores for the treatment of MI. Li et al. (Zulkifli et al., 2023) demonstrated that elesclomol alleviated Cu deficiency in the yeast, mouse and zebrafish models through Cu delivery to mitochondria and the restoration of CcO function. Despite the obtained findings, the exact mechanism of elesclomol in regulating Cu delivery in cells is still largely unclear.

Conventional Cu carriers face limitations concerning versatility and targeted delivery. Ineffective regulation of Cu transport can result in excessive Cu supplementation and Fenton-like reactions, leading to oxidative damage (Yuan et al., 2017). Due to accumulation and subsequent oxidative stress, non-specific Cu delivery may cause tissue damage. To address these issues, Su et al. (2019) proposed the targeted ion carrier–based metal supplements (TIMS) concept, an approach for site-specific metal delivery within organisms. Considering these considerations, Cu ionophores can provide the promising treatment for target Cu-mediated cell death during MI.

In clinical studies, serum Cu contents significantly increase among MI patients in relative to healthy individuals (Yesmin et al., 2016b; Begum et al., 2023a). Substantial clinical evidence has suggested that Cu can be a valuable target for predicting, treating, and assessing the prognosis of cardiovascular diseases (Wang et al., 2023; Yang L. et al., 2023). Propensity score-matched analyses indicate that the higher dietary Cu intake is associated with a reduced risk of MI, especially among elderly women, overweight individuals, smokers, and those with hypertension or diabetes (Wen et al., 2022). He and James Kang, (2013) found that myocardial ischemia usually resulted in the decreased Cu content in the heart, and Cu supplementation could enhance HIF-1 transcriptional activity, therefore restoring the angiogenic capacity and increasing capillary density in the heart. This finding has implications for the development of the association between elevated serum Cu levels and MI, though subgroup analysis indicated considerable effect modification by ethnicity. Despite the correlation between Cu and MI, further mechanistic research is required to demonstrate these findings.

## 5 Cuproptosis-related genes

**FIGURE 2:**
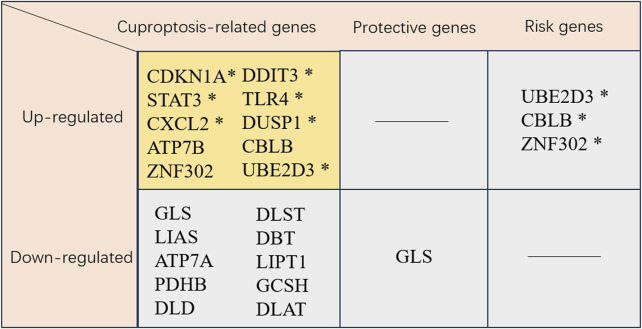
Cuproptosis-related genes. * This gene has been validated. CDKN1A, cyclin-dependent kinase inhibitor 1A; DDIT3, DNA damage inducible transcript 3; STAT3, signal transducer and activator of transcription 3; TLR4, toll like receptor 4; CXCL2, C-X-C motif chemokine ligand 2; DUSP1, dual specificity phosphatase 1; ATP7B, ATPase copper transporter 7B; CBLB, Cbl proto-oncogene B; ZNF302, zinc finger protein 302; Ube2d3, ubiquitin-conjugating enzyme E2D 3; GLS, glutaminase; DLST, dihydrolipoamide S-succinyltransferase; LIAS, lipoic acid synthetase; DBT, dihydrobiopterin; ATP7A, ATPase copper transporter 7A; LIPT1, lipoyltransferase 1; PDHB, pyruvate dehydrogenase E1 subunit beta; GCSH, glycine cleavage system protein H; DLD, dihydrolipoamide dehydrogenase; DLAT, dihydrolipoamide S-acetyltransferase.

Cuproptosis is the novel cell death type dependent on mitochondrial respiration. Nevertheless, research concerning the impact of cuproptosis-related genes on MI is limited. Recently, researchers have systematically evaluated the genetic alterations in MI based on bioinformatics approaches. This study reviews cuproptosis genes and targets potentially associated with MI, aiming to provide insights for the improved diagnosis and treatment of the disease (Figure 2).

Liu et al. (2022) indicated that the cuproptosis-related gene GLS was likely associated with MI development through the HIF-1 pathway. Notably, GLS was found to be correlated with immune-related pathways, including the chemokine and T-cell receptor pathways. In addition, GLS participated in hypoxia-related pathways, including the HIF-1 pathway, consistent with KEGG analysis. This dual involvement in immune and hypoxic signaling positions GLS as a promising biomarker for MI, with its expression potentially reflecting both ischemic stress and inflammatory burden. As a therapeutic target, modulating GLS activity may offer a dual strategy to alleviate myocardial hypoxia and regulate immune cell infiltration, particularly given its negative correlation with monocyte abundance in acute coronary syndrome (Liu et al., 2022).

Using bioinformatics analysis, Miao et al. (2023) identified six immune-related genes (CXCL2, DDIT3, DUSP1, CDKN1A, TLR4, and STAT3) associated with cuproptosis and ferroptosis as the candidate early diagnostic biomarkers for MI. Based on ROC curve analysis, they were of prominent prediction significance. Peripheral blood samples in MI patients and acute myocardial infarction (AMI) mouse model verified these six genes as candidate biomarkers, highlighting their diagnostic utility. These genes may also serve as therapeutic targets, with curcumin and N-acetyl-L-cysteine identified as potential modulators that could intervene in cuproptosis, ferroptosis, and immune infiltration pathways to improve MI outcomes (Miao et al., 2023). Yang et al. (2024) identified UBE2D3 as a key gene in MI development through promoting cuproptosis, leading to cardiomyocyte death. As an E2 family member, UBE2D3 was highly expressed in MI model animals and OGD-treated cardiomyocytes, with its expression correlated with ischemic injury. In myocardial ischemia/reperfusion injury, UBE2D3 enhanced p62 ubiquitination to exacerbate autophagic flux impairment (Wang et al., 2021). Preclinical studies showed that UBE2D3 knockdown improved cell viability and decreased LDH release in OGD models. Notably, its association with neutrophil infiltration highlighted UBE2D3 as a dual regulator of inflammation and cell death, positioning it as a promising therapeutic target for MI intervention (Wang et al., 2021). According to the MI-cuproptosis differentially expressed genes risk model and drug prediction in the Coremine Medical database, Zhang et al. (Fang C. et al., 2024) demonstrated the downregulation of nine cuproptosis-related genes (DLST, LIAS, DBT, ATP7A, LIPT1, PDHB, GCSH, DLD, and DLAT), whereas the upregulation of one gene (ATP7B) in MI patients. These ten cuproptosis-related genes demonstrated significant associations with immune cell infiltration. Recently, the application of machine learning models to predict MI prevalence based on demographic and imaging data has gained prominence. For instance, Wang et al. (2024) identified two key cuproptosis-related genes, ZNF302 and CBLB, through these models, which are vital for MI diagnosis and treatment. A GWAS analysis of congenital heart disease revealed ZNF302 as the significant differential gene (Jiang et al., 2018), suggesting its key role in cardiac development. Furthermore, a study highlighted the potential of differentially expressed genes, metabolites, and microbiota in MI regulation following CBLB intervention, demonstrating its vital role in MI regulation (You et al., 2024). Wang et al. (2024) also found that MI mice exhibited significant MI and impaired cardiac function, with an obvious increase in CBLB and ZNF302 expression in the myocardium, underscoring the importance of these genes in MI diagnosis and treatment. Zhou et al. (2024b) used nine mainstream machine learning algorithms and stacking methods to develop robust AI models for MI diagnosis. Their findings suggested that SLC31A1 might be a promising emerging diagnostic biomarker for MI. They confirmed the vital role of SLC31A1 expression in the MI immune milieu and its potential to enhance cardiac tissue recovery post-MI using animal models, therefore emphasizing its prospective utility as a diagnostic biomarker. Evidence has indicated that CTR1 (also known as SLC31A1) is the redox sensor for promoting angiogenesis in endothelial cells. Mechanistically, CTR1 is under rapid sulfenylation at Cys189 at the cytosolic C terminal following VEGF stimulation, inducing the generation of the CTR1-vascular endothelial growth factor receptor type 2 (VEGFR2) disulfide bond and the co-internalization into endosomes, thus triggering the persistent VEGFR2 signal transduction and enhancing angiogenesis (A et al., 2022).

## 6 Conclusion and perspectives

Despite significant advancements in modern medicine, MI is a major challenge in both medical care and public health. Obviously, there is a concerning trend towards younger age groups among patients with MI (Arora et al., 2019). Moreover, readmission rates, mortality, and hospitalization burdens for MI patients have not decreased (Chen P. et al., 2021; Tisminetzky et al., 2021; Alves et al., 2022). Recent research has increasingly performed to explore Cu-related cell death mechanisms in cancers, cardiovascular diseases, and additional conditions. It is urgently needed to identify novel targets and strategies for the treatment and prevention of MI. Based on the studies on MI, the high serum Cu level is significantly associated with MI. Cu overload influences mitochondrial function and exacerbates the development of MI (Muñoz-Bravo et al., 2023). Studies have reported that Cu ions contribute to aberrant lipoylated protein aggregation, leading to the downregulated Fe-S cluster protein level, which induces proteotoxic stress, finally causing cell death. In addition, Cu affects cell death through mechanisms involving ROS and inflammatory responses. Furthermore, the role of Cu-induced cell death in linking oxidative stress with inflammation underscores its significance in MI pathogenic mechanism. Therefore, it can be hypothesized that cuproptosis is a novel therapeutic target for MI. Dysregulation of Cu metabolism can alter cardiac gene expression, and Cu supplementation has been found to mitigate certain unfavorable cardiac outcomes caused by Cu deficiency. Given the association of Cu homeostasis with various disease states, further research plays an essential role in clarifying how Cu imbalance leads to cellular damage. In summary, targeting cuproptosis may provide the promising method to the diagnosis, treatment, prevention and assessment of MI.

## Glossary

AMI: acute myocardial infarction
ATOX1: Antioxidant 1
ATP7A: ATPase copper transporter 7A
ATP7B: ATPase copper transporter 7B
CBLB: Cbl proto-oncogene B
CcO: Cytochrome c oxidase
CDKN1A: Cyclin-dependent kinase inhibitor 1A
Cp: Ceruloplasmin
CRIP2: Cysteine-rich protein 2
Cu: Copper
CuBPs: Copper-binding proteins
CuCe: NPs Cu-deposited ceria nanoparticles
CXCL2: C-X-C motif chemokine ligand 2
DBT: Dihydrobiopterin trihydrobiopterin synthase
DDIT3: DNA damage inducible transcript 3
DLAT: Dihydrolipoamide S-acetyltransferase
DLD: Dihydrolipoamide dehydrogenase
DLST: Dihydrolipoamide S-succinyltransferase
DUSP1: Dual specificity phosphatase 1
EDTA: Ethylenediaminetetraacetic acid disodium salt
Fe–S: Iron–sulfur
GCSH: Glycine cleavage system protein H
GLS: Glutaminase
GSH: Glutathione
GSSH: Oxidized glutathione
HIF-1: Hypoxia-inducible factor 1
I/R: Ischemia/reperfusion
LDL: Low-density lipoprotein
LIAS: Lipoic acid synthetase
LIPT1: Lipoyltransferase 1
MI: Myocardial infarction
MMP-2: Matrix metalloproteinase 2
PDHB: Pyruvate dehydrogenase E1 subunit beta
PF: Paeoniflorin
PGC1-α: Peroxisome proliferator-activated receptor gamma coactivator 1-alpha
ROS: Reactive oxygen species
SOD: Superoxide dismutase
STAT3: Signal transducer and activator of transcription 3
TCA: Tricarboxylic acid
TGF-β: Transforming growth factor-β
TIMS: Targeted ion carrier–based metal supplements
TLR4: Toll like receptor 4
TM: Tetrathiomolybdate
UBE2D3: Ubiquitin-conjugating enzyme E2D 3
VEGF: Vascular endothelial growth factor
VEGFR2: Vascular endothelial growth factor receptor type 2
ZNF302: Zinc finger protein 302
α-SMA: α-smooth muscle actin
